# Complete mitochondrial genome sequence for the Taiwan Blue Magpie *Urocissa caerulea* (Passeriformes: Corvidae)

**DOI:** 10.1080/23802359.2018.1481778

**Published:** 2018-06-07

**Authors:** Hsin-I Hsieh, Hsuan-Yi Hou, Rui-Xuan Chang, Yi-Ning Cheng, Nian-Hong Jang-Liaw

**Affiliations:** a Conservation and Research Center, Taipei Zoo, Taipei, Taiwan;; b Department of Animal Science, National Chiayi University, Chiayi, Taiwan;; c Department of Animal Science, National Pingtung University of Science and Technology, Pingtung, Taiwan

**Keywords:** Complete mitochondrial genome, Taiwan Blue Magpie, *Urocissa caerulea*

## Abstract

Taiwan Blue Magpie (*Urocissa caerulea*) is endemic to Taiwan and listed as threatened species protected by law. In this study, we first determined and described the complete mitochondrial genome of Taiwan Blue Magpie. The circle genome is 16,928 bp in length, and contains 13 protein coding, 22 tRNA, two rRNA genes, and one non-coding control region (CR). The overall base composition of the mitochondrial DNA is 30.99% for A, 24.69% for T, 30.07% for C, and 14.25% for G. The percentage of G + C content is 44.32%. This work provides fundamental molecular data which will be useful for evolution and phylogeny studies on Corvidae in the future.

*Urocissa* is a genus of birds in the family Corvidae, which includes six species distributed in Indian subcontinent, Indochina, Southern China, and Taiwan (BirdLife International 2016; Integrated Taxonomic Information System 2018). Though members of genus *Urocissa* have a wide distribution range and are appealing for their appearances, little phylogeny research information such as sequence data are available at present. Here, a complete mitochondrial genome of Taiwan Blue Magpie is presented. The Taiwan Blue Magpie (*Urocissa caerulea*) is an endemic species in Taiwan, which lives in broadleaf forests or parks with abundant trees from lowland to mid-elevation. The material we used in this study was the health-examination blood sample kept in Wildlife Cryobank, Taipei Zoo (deposit no. 20170210D10). The voucher of blood sample is D1818, a male Taiwan Blue Magpie transported from Hsinchu Zoo on 3 July 2017, and is kept in Taipei Zoo currently. The usage of this blood sample was authorized by Forestry Bureau, Council of Agriculture, Executive Yuan, Taiwan, with the permission document number 1061700466 (2017-19). Genomic DNA was extracted using the DNeasy Blood & Tissue Kit (QIAGEN, Hilden, Germany). The PCR and sequencing experiments were carried out mainly by the following procedures described by Chiang et al. (2017). We designed two pairs of long PCR primers in the beginning (TBM-1F: 5′-CATCATCTGAGAGGCTTTCGCATCC3-3′ and TBM-1R: 5′-CTCAGGCTCATTCTACTAGTGTTTGTC-3′; TBM-2F: 5′-AACAAAGAGACCTGAAACATCGGAGTA-3′, and TBM-2R: 5′-AATGTGGTGTTGAGGTTGCGGTCTGTT-3′) based on the sequences of a sibling species, Red-billed Blue Magpie (*U. erythrorhyncha*; Genbank accession no. JQ423932). Two parts of long mtDNA sequence were sequenced separately by primer walking on an ABI 3730 DNA Analyzer (Applied Biosystems, Foster, CA). It took 26 primer pairs to complete the primer walking procedure, and the sequenced fragments were assembled into the whole mitochondrial genome sequence with the aid of SeqMan version 7.1 (DNAStar, Madison, WI) and then adjusted manually by eye. The whole mitochondrial genome sequence was deposited in GenBank with the accession number MG932654.

The organization of mitochondrial genome of D1818 is determined to be 16,928 bp in size, including 13 typical vertebrate protein-coding genes, 22 transfer RNA genes, 2 ribosomal RNA genes, and a control region (CR). All genes were encoded on the H-strand with the exception of one protein-coding gene (*ND6*) and eight tRNA genes [*tRNA^Gln^*, *tRNA^Ala^*, *tRNA^Asn^*, *tRNA^Cys^*, *tRNA^Tyr^*, *tRNA^Ser(UCN)^*, *tRNA^Pro^*, and *tRNA^Glu^*]. The base composition was counted using MEGA7 (Kumar et al. 2016). The overall base composition in descending order is: A (30.99%), T (24.697%), C (30.07%), G (14.25%) with 44.32% GC content. The positions of RNA genes were predicted by the MITOS (Bernt et al. 2013) and the locations of protein-coding genes were identified by comparing with the homologous gene of Red-billed Blue Magpie. The 22 tRNA genes range from 69 to 75 bp in length and all can fold into a typical cloverleaf secondary structure that was estimated by the online software tRNAscan-SE v2 (Lowe and Chan 2016). The two ribosomal RNA genes, *12S rRNA* (947 bp) and *16S rRNA* (1602 bp), were located between *tRNA^Phe^* and *tRNA^Leu(UUR)^* genes and were separated by the *tRNA^Val^* gene as seen in other vertebrates. Except for *COX1* and *ND4L*, the remaining 11 protein-coding genes start with an ATG codon. Eleven protein-coding genes end with complete stop codons TAA (*ND2*, *COX2*, *ATP8*, *ATP6*, ND3, *ND4L, CYTB,* and *ND6*), AGA (*ND1* and *ND5*), and AGG (*COX1*). The remaining protein-coding genes end with the incomplete stop codons represented by ‘T’ (*COX3* and *ND4*). CR is 1347 bp long. No tandem repeat segment was found, checked by online software ‘TANDEM REPEATS FINDER’ (Benson 1999). Similar to other Aves’ mitochondrial genome organization, the gene arrangement between *ND5* and CR is *CYTB*- *tRNA^Thr^*- *tRNA^Pro^*- *ND6*- *tRNA^Glu^*(e.g. Härlid et al. 1998; Lee et al. 2017; Ludwig et al. 2017), which is different from that of mammals and fish (*ND6*- *tRNA^Glu^* -*CYTB*- *tRNA^Thr^*- *tRNA^Pro^*; e.g. Xu et al. 1996; Chang et al. 2013, 2016; Jang-Liaw et al. 2013; Chiang et al. 2017; Hou et al. 2018), as well as the absence of the origin of L-strand replication (O_L_), which is common in other vertebrates’ mitochondrial genome. As shown in the Neighbor-Joining (NJ) analysis, which included mitochondrial genome of *U. caerulea* and the other 10 species from the family Corvidae using *Emberiza sulphurata* (Passeriformes) of family Fringillidae as an outgroup (Figure 1), *U. caerulea* clustered with another *Urocissa* species, *U. erythrorhyncha*, and is highly supported by a bootstrap value of 100. Thus, the newly determined mitochondrial genome will enrich the basic molecular data of *Urocissa*.

**Figure 1.**
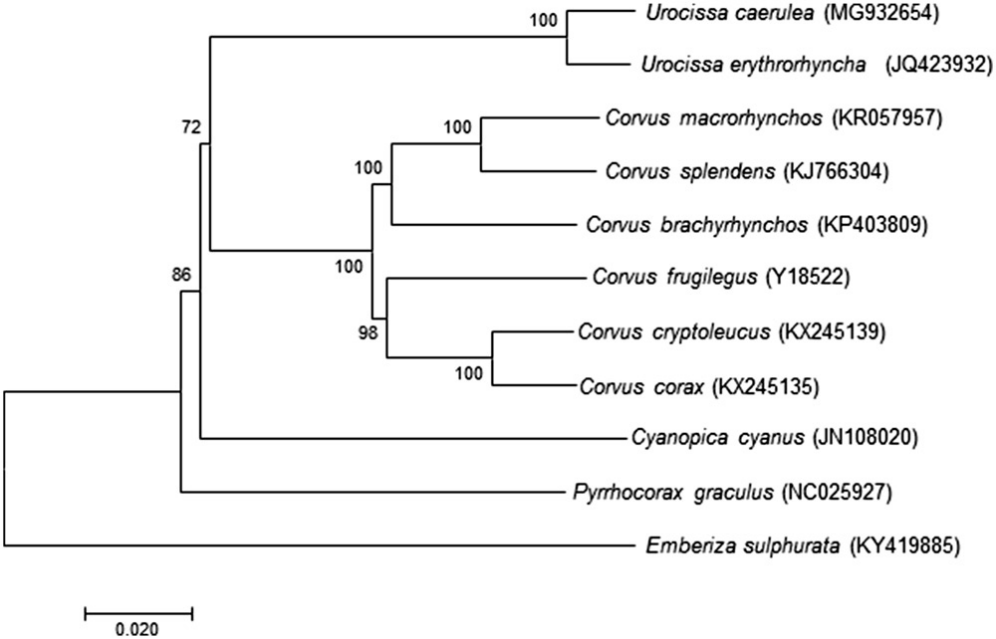
Neighbor-Joining (NJ) phylogenetic tree of *U. caerulea* and the other 10 species using *Emberiza sulphurata* as an outgroup were conducted in MEGA7. The percentage of replicate trees in which the associated taxa clustered together in the bootstrap test (1000 replicates) are shown next to the branches. The evolutionary distances were computed using the Kimura 2-parameter method and are in the units of the number of base substitutions per site. All species’ accession numbers are listed behind taxa.
